# Fine Tuning of Ca_V_1.3 Ca^2+^ Channel Properties in Adult Inner Hair Cells Positioned in the Most Sensitive Region of the Gerbil Cochlea

**DOI:** 10.1371/journal.pone.0113750

**Published:** 2014-11-19

**Authors:** Valeria Zampini, Stuart L. Johnson, Christoph Franz, Marlies Knipper, Matthew C. Holley, Jacopo Magistretti, Giancarlo Russo, Walter Marcotti, Sergio Masetto

**Affiliations:** 1 Department of Biomedical Science, University of Sheffield, Sheffield, United Kingdom; 2 Department of Otolaryngology, Tübingen Hearing Research Centre, Laboratory of Molecular Physiology of Hearing, University of Tübingen, Tübingen, Germany; 3 Department of Biology and Biotechnology “Lazzaro Spallanzani”, University of Pavia, Pavia, Italy; 4 Department of Brain and Behavioral Sciences, University of Pavia, Pavia, Italy; University of South Florida, United States of America

## Abstract

Hearing relies on faithful signal transmission by cochlear inner hair cells (IHCs) onto auditory fibres over a wide frequency and intensity range. Exocytosis at IHC ribbon synapses is triggered by Ca^2+^ inflow through Ca_V_1.3 (L-type) Ca^2+^ channels. We investigated the macroscopic (whole-cell) and elementary (cell-attached) properties of Ca^2+^ currents in IHCs positioned at the middle turn (frequency ∼2 kHz) of the adult gerbil cochlea, which is their most sensitive hearing region. Using near physiological recordings conditions (body temperature and a Na^+^ based extracellular solution), we found that the macroscopic Ca^2+^ current activates and deactivates very rapidly (time constant below 1 ms) and inactivates slowly and only partially. Single-channel recordings showed an elementary conductance of 15 pS, a sub-ms latency to first opening, and a very low steady-state open probability (*P*_o_: 0.024 in response to 500-ms depolarizing steps at ∼−18 mV). The value of *P*_o_ was significantly larger (0.06) in the first 40 ms of membrane depolarization, which corresponds to the time when most Ca^2+^ channel openings occurred clustered in bursts (mean burst duration: 19 ms). Both the *P*_o_ and the mean burst duration were smaller than those previously reported in high-frequency basal IHCs. Finally, we found that middle turn IHCs are likely to express about 4 times more Ca^2+^ channels per ribbon than basal cells. We propose that middle-turn IHCs finely-tune Ca_V_1.3 Ca^2+^ channel gating in order to provide reliable information upon timing and intensity of lower-frequency sounds.

## Introduction

In mammals, inner hair cells (IHCs) are responsible for the transduction of sound stimuli into an electrical signal, which is sent to the brain by afferent fibres. Sound stimuli entering the cochlea cause the mechanical displacement of IHC stereocilia, which leads to the flow of a cation current into hair cells via mechanoelectrical transducer channels. This depolarizing current activates Ca_V_1.3 Ca^2+^ channels [1], [2] at the IHC active zones [3], [4] thus inducing Ca^2+^-dependent exocytosis [5], [6], [7] of glutamate that activates afferent fibres [8]. Each afferent fibre, which makes only one synaptic contact with an adult IHC [9], receives information from a single presynaptic specialization named the synaptic ribbon [10]. Therefore it is essential that sound stimuli encoded by the IHC are accurately preserved at this initial stage.

Sound frequencies are encoded in terms of the topography of the stimulated cell along the cochlea (so-called tonotopicity). For high-frequency tones (basal cochlear turn in the mouse and gerbil), IHC membrane filtering prevents their membrane potential tuning to stimulus frequencies above ∼3.5 kHz. Therefore, the receptor potential of basal IHCs is graded and sustained (d.c. component) [11] and supported by the maintained activity of presynaptic Ca^2+^ channels [4], which activate rapidly to accurately signal high-frequency sound onset [12]. IHCs and afferent nerve fibres in the lower-frequency regions (apical and middle cochlear turns in gerbils: < a few kHz) show tuning and phase-locking to sound stimulation [11], enabling a time code of frequency to allow frequency discrimination and sound localization based on the detection of interaural time differences [13], [14]. This requires the IHC receptor potentials not only to be graded to sound intensity, similar to basal cells, but also have a phasic (a.c.) component representing the sound frequency [15]. As such, Ca^2+^ channels in low-frequency IHCs would need to activate/deactivate faithfully during the excitatory/inhibitory phase of the sensory signal. In this study we investigated Ca^2+^ channels in IHCs positioned at the middle turn (frequency ∼2 kHz) of the adult gerbil cochlea, which is their most sensitive hearing region [16]. This cochlear region represents a close match with the frequency range present in the apical coil of the more commonly used mouse (∼3 kHz [17]).

We found that most of the properties of Ca_V_1.3 Ca^2+^ channels in IHCs, i.e. the elementary conductance, the sub-ms activation kinetics, the rapid deactivation, the burst opening modality and the slow inactivation, are similar between high- (basal: ∼30 kHz: [4]) and low-frequency (middle: ∼2 kHz) IHCs. However, Ca^2+^ channels in IHCs positioned in the 2 kHz region showed briefer bursts of activity in response to sustained depolarizations and an overall lower *P*_o_ compared to those in basal high-frequency IHCs.

As a result of the Ca^2+^ channel properties found here, low-frequency sound waves of increasing intensity would elicit phase-locked Ca^2+^ influx at progressively more presynaptic active zones, allowing for internal coding of both frequency and intensity properties of sounds within the middle coil characteristic frequency range.

## Results

### Ca^2+^ channel expression in IHCs of the middle gerbil cochlear turn

**Figure 1:**
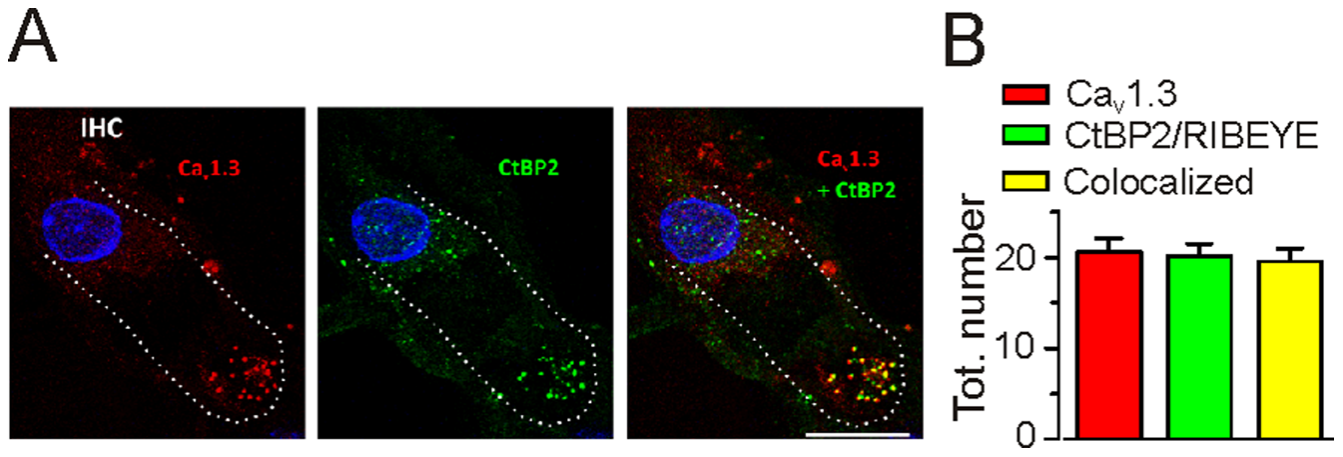
Distribution of Ca_V_1.3 and CtBP2/RIBEYE in adult gerbil IHCs. **A**, IHC positioned in the middle coil (frequency ∼2 kHz) of the adult P20 gerbil cochlea immunostained for the Ca_V_1.3 Ca^2+^ channel (red) and ribbon marker CtBP2/RIBEYE (green). Note that both Ca_V_1.3 Ca^2+^ channels and ribbons are localized at the IHC basal pole, which is the region used for all single Ca^2+^ channel recordings shown in the following figures. Merged images are shown in the right column, which show colocalization between CtBP2/RIBEYE and Ca^2+^ channel immunopositive spots in yellow. White dotted lines delineate the IHC. Images represent the maximum intensity projection over all layers of the z-stack. Nuclei were stained with DAPI (blue). Scale bar = 10 µm. **B**, Total number of immunopositive spots for Ca_V_1.3 (red bar), for CtBP2/RIBEYE (green bar) and colocalized (yellow bar); n = 13 IHCs analyzed from three gerbils.

The distribution of Ca_V_1.3 channels within IHCs of the gerbil cochlear middle turn was investigated by performing immunolabelling experiments (Figure 1). Ca^2+^ channel clusters in adult IHCs were only found at their presynaptic region (Figure 1A: for mice see also [5], [18], [19]). The average number of immunopositive Ca_V_1.3 spots was 20.6±1.5 (*n* = 13), which co-localized with synaptic ribbons (Figure 1B). Single Ca^2+^ channel recordings were only performed from the basal pole of IHCs.

### Unitary current and open probability of Ca_V_1.3 Ca^2+^ channels in middle coil IHCs

Single Ca^2+^ channel recordings from IHCs were performed from acutely isolated organs of Corti maintained at body temperature, using 5 mM extracellular Ca^2+^ and 5 µM BayK 8644 in the recording pipette. IHCs were held at their resting membrane potential (i.e. the patch was clamped at 0 mV), which was then stepped to different test potentials. The use of BayK 8644 was essential when working at body temperature since in its absence the majority of single channel openings were not resolved (see also [18]). BayK 8644 is known to produce longer openings of L-type Ca^2+^ channel [20], [21], [22], [23]. Previous investigation on mouse IHCs have shown that at macroscopic level the impact of BayK 8644 was to increase the peak Ca^2+^ current with no change in activation kinetics [18].

**Figure 2:**
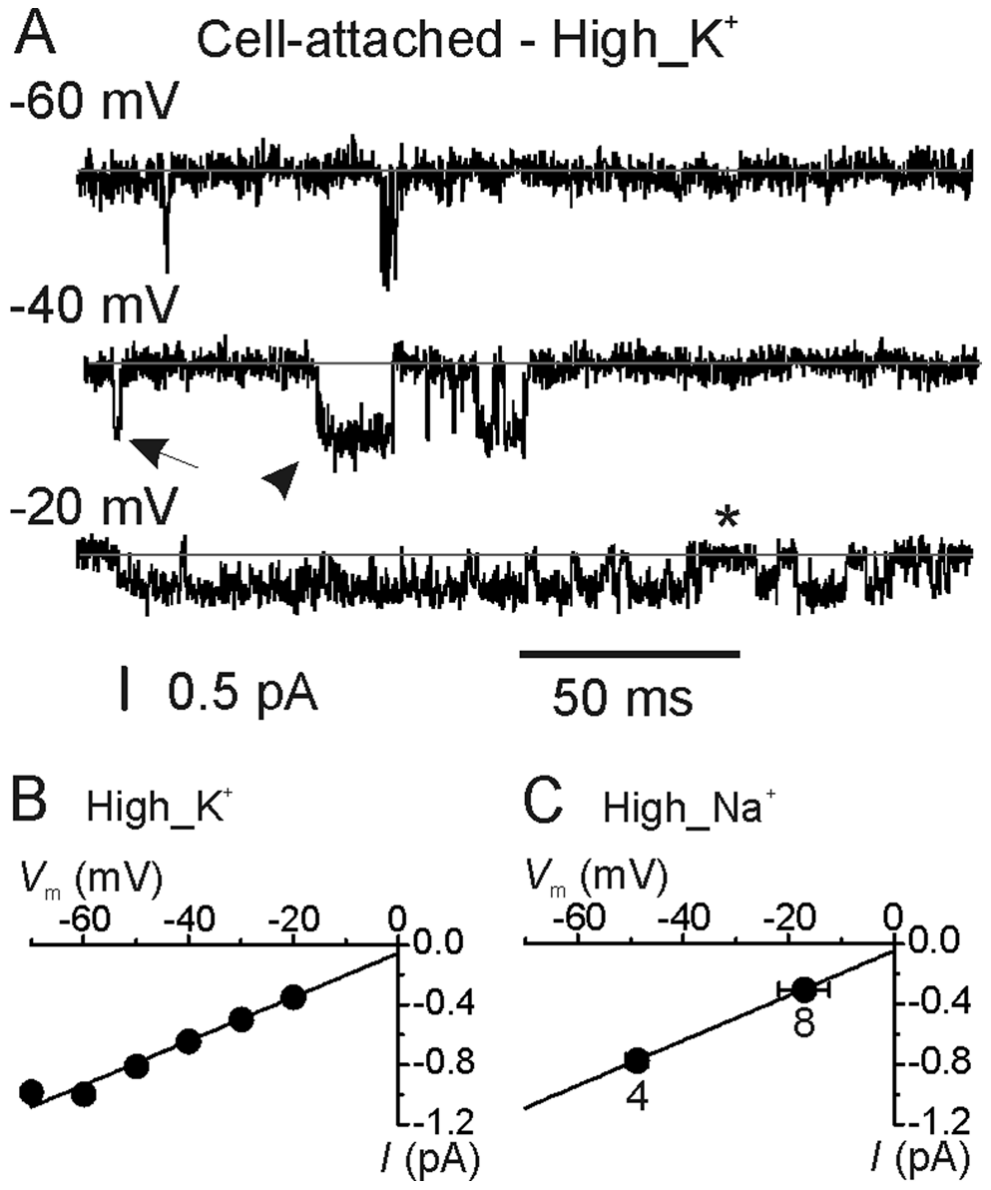
Elementary Ca^2+^ currents recorded in cell-attached configuration. **A**, Representative unitary currents recorded from adult IHCs positioned in the middle region of the gerbil cochlea using a high-K^+^ extracellular solution (High_K^+^; see Methods). The pipette solution contained 5 mM Ca^2+^ and 5 µM BayK 8644. Transmembrane patch potentials are shown next to the traces. The inter-stimulus interval in all cell-attached recordings was 2 s. Grey lines indicate the channel closed state (here and in the other figures Ca^2+^ channel openings are indicated by a downward deflection of the current trace). Arrows and arrowheads show single brief and long-lasting openings, respectively. In several cases clusters of openings were seen, which were interrupted by short closing periods (asterisk). **B**, Average current-voltage (*I*-*V*) data for single Ca^2+^ channel currents recorded in High_K^+^ from eight IHCs (4≤*n*≤8 patches). Mean channel conductance values was 14.9±0.1 pS. **C**, Average single Ca^2+^ channel recordings from IHCs in high_Na^+^ extracellular solution (High_Na^+^; see Methods) plotted on the fit from panel B. Number of IHCs tested is shown. Note that the slope of the linear fit was virtually identical to that shown in panel **B**.

Using a high-K^+^ extracellular solution, which allowed control over the transmembrane potential in the recorded patches (see Methods), we found that the unitary Ca^2+^ channel openings became more frequent and longer with membrane depolarization (Figure 2A). Openings were often very brief (Figure 2A, arrow), although some longer openings were occasionally seen (Figure 2A, arrowhead). In several cases we recorded clusters of Ca^2+^ channel openings that were interrupted by brief closing periods (asterisk in Figure 2A). We defined “burst” as a cluster of openings separated by brief closures (see also below). This gating behavior resembles mode 1 and 2 originally reported for high-voltage-activated (HVA) Ca^2+^ channels, also named long-lasting (L-Type) Ca^2+^ channels because of their depolarized activation voltage threshold (∼−30 mV) and little inactivation [21], [23]. However, only in recent years it has been shown that Ca_V_1.3 channels activate at more negative voltages (∼−60 mV) than the other members of the HVA family (1.1, 1.2, and 1.4) [24], [25], [26], [27]; for a recent review see [28]. Indeed, single Ca_V_1.3 Ca^2+^ channel activity was present at the resting membrane potential of adult gerbil IHCs (near −60 mV). The single channel current-voltage (*I-V*) relation was linear over the voltage range investigated, with an average slope conductance of 15 pS (Figure 2B).

**Figure 3:**
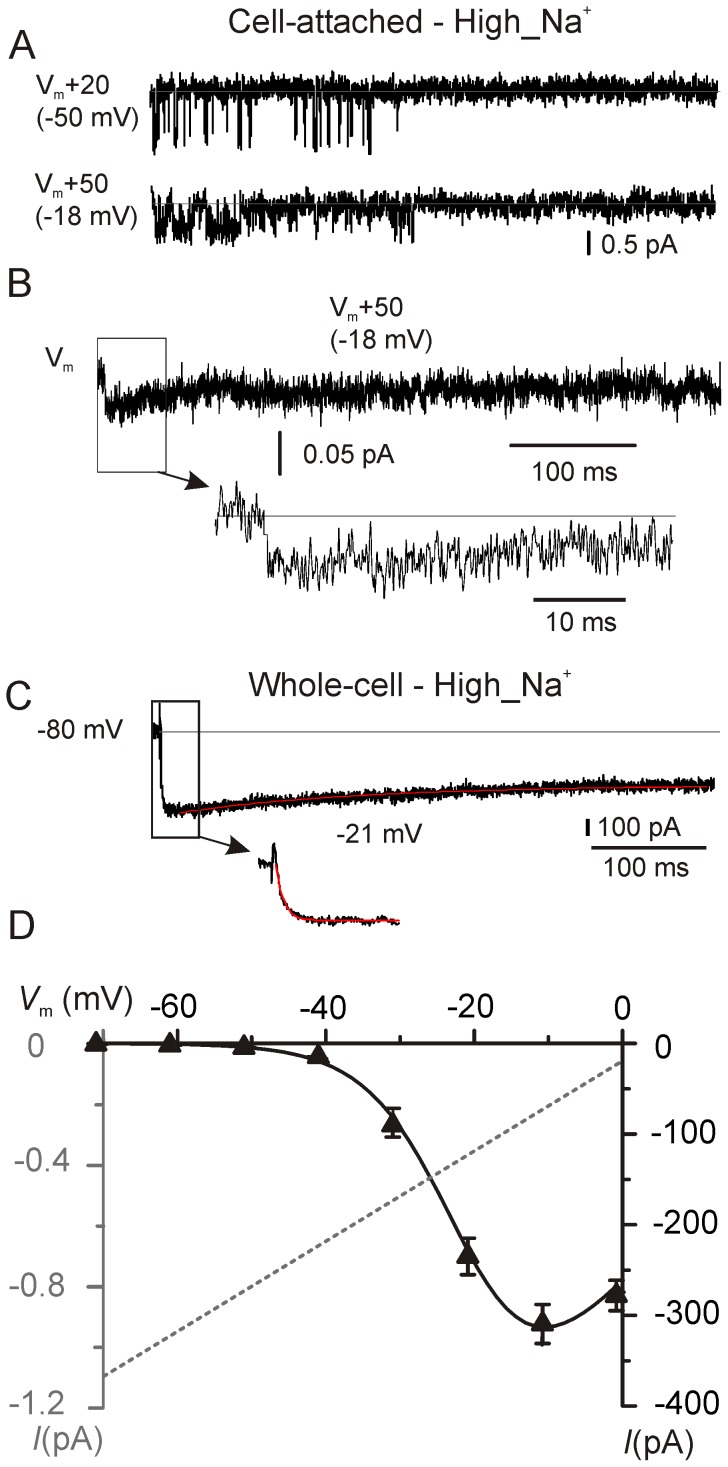
Elementary and macroscopic Ca^2+^ currents recorded in high_Na^+^ extracellular solution. **A**, Representative unitary currents recorded from middle-coil IHCs in high_Na^+^ extracellular solution (see Methods) with 5 mM Ca^2+^ and 5 µM BayK 8644. Values for transmembrane patch potentials (*V*_m_+20 and *V*_m_+50) represent the sum of the voltage step command (+20 mV or +50 mV) and the cell resting membrane potential (*V*_m_). The actual transmembrane voltage (∼−50 mV and ∼−18 mV) was derived from the single channel current amplitude of Figure 2C. Continuous gray lines indicate the channel closed state. **B**, Ensemble-averaged Ca^2+^ current at Vm+50 mV (data from 163 active sweeps from 8 patches). Ca^2+^ current activation time course is shown in an expanded scale below the trace. **C**, Representative macroscopic *I*_Ca_ recorded from a middle-coil IHC in the whole-cell configuration. Recordings were performed using the same extracellular solution to that for the single-channel recordings and at the cell membrane voltage of −21 mV, starting from a holding voltage of −80 mV. Activation and inactivation time course are fitted using a single exponential. The horizontal dashed line in **B** and **C** indicates the zero current level. **D**, Mean current-voltage relationship for the macroscopic Ca^2+^ current recorded in high_Na^+^ extracellular solution from 6 adult gerbil middle turn IHCs. Data points were fitted with eqn. 3. The fitting parameters are: *g*_max_ 9.6 nS (±1.1); *V*_rev_ 28.1 mV (±2.0); *V*_1/2_ −20.6 mV (±2.02); S 5.9 mV (±0.5). The current-voltage relationship for the elementary current (same as Figure 2C) is also shown in gray.

In order to study the Ca^2+^ channel behaviour while maintaining adult IHCs at their physiological membrane potential, all the following experiments were performed with the high_Na^+^ extracellular solution in the bath. The use of this extracellular solution prevented us from directly determining the IHC resting membrane potential. Therefore, the patch transmembrane voltage is indicated as the unknown IHC membrane potential (*V*_m_) plus the voltage step delivered to the patch pipette (e.g. *V*_m_+20: 20 mV depolarization from *V*_m_). We were able to estimate the patch transmembrane voltage using the amplitude of the elementary Ca^2+^ current and extrapolating it from the *I*-*V* curves in high_K^+^ extracellular solution, assuming identical single channel conductance between the two recording conditions (Figure 2C; see also [4]). Examples of single Ca^2+^ channel recordings in high_Na^+^ extracellular solution obtained at *V*_m_+20 and *V*_m_+50 are shown in Figure 3A. The estimated mean transmembrane voltage applied to IHCs was −50 mV for *V*_m_+20 and −18 mV for *V*_m_+50. Single Ca^2+^ channel open probability (*P*_o_) increased with depolarization (Figure 3A, lower trace). However, the percentage of null-sweeps (sweeps in response to 500 ms voltage steps with no detectable channel activity) was rather high even at *V*_m_+50 mV (55%; 294/535; n = 6 cells). As previously noted for basal IHCs [4], null sweeps were often clustered, indicating that the channel could shift from a willing-to-open to an unwilling-to-open configuration, possibly due to the biochemical state of the cell [23], [29], [30]. The possibility that the high frequency of null sweeps was due to a high incidence of brief, unresolved single channel openings missed during threshold analysis of amplitude levels was excluded based on the fact that we found no significant difference in the variance and the standard deviation of the current recorded in null sweeps at *V*_m_+20 and at *V*_m_+50.

When considering the active sweeps only (sweeps containing at least one Ca^2+^ channel opening during the 500 ms stimulus), *P*_o_ was 0.009 (±0.022) at V_m_+20 mV (n = 7 patches) and 0.024 (±0.048) at *V*_m_+50 mV (n = 6 patches), the latter value being much smaller than found in adult basal gerbil IHCs (0.21; [4]). However, when only the first 40 ms of the sweeps were considered, *P*_o_ at *V*_m_+50 mV increased to 0.058 (±0.116; P<0.001). Thus, although the channel shows a very low overall opening probability during prolonged depolarizations, it is more inclined to remain open at the beginning of a stimulus than afterwards. Visual inspection of the recordings revealed a tendency of channel openings to cluster in the initial part of the sweeps (Figure 3A; see also below). The activation time course of the ensemble-average current (Figure 3B) could not be fully resolved because it already reached the peak at the earliest time points available, which indicates a sub-ms activation kinetics (Figure 3B, inset). The inactivation kinetics was also difficult to assess due to the relatively high current noise and was found to vary between 30 ms and 180 ms depending on the starting position of the fit with a monoexponential function (not shown). The steady-to-peak ratio of the ensemble-average current (40%) was quite consistent with the ratio (35%) of the average *P*_o_ measured in the first 40 ms (0.06) to that measured in the last 100 ms (0.021±0.059) of the sweeps (total sweeps number: 294 from 8 patches; total duration of each sweep: 500 ms).

### Macroscopic Ca^2+^ current in IHCs of the middle cochlear region

We performed whole-cell Ca^2+^ current (*I*_Ca_) recordings from age- and location-matched gerbil IHCs using extracellular solutions analogous to those used for cell-attached recordings (high_Na^+^ extracellular solution with 5 mM Ca^2+^ together with 100 µM linopirdine, 50 µM niflumic acid and 5 µM BayK 8644). The partial inactivation of the macroscopic *I*_Ca_ (Figure 3C) at −21 mV was consistent with the decrease in single Ca^2+^ channel *P*_o_ during the sweeps (Figure 3A) and the partial inactivation of the ensemble-average current (Figure 3B) in cell-attached recordings, although the steady-to-peak ratio of the macroscopic current was larger (65%). Fitting the inactivation kinetics with a single exponential function provided an inactivation time constant, τ_i_, of 181 ms (±13 ms; *n* = 6). Thus, Ca^2+^ channel inactivation appears to be more pronounced and faster than that measured in cell-attached configuration, possibly due to the fact that the whole-cell configuration could affect the normal intracellular modulation of the Ca^2+^ channel or the physiological ATP concentration (see Discussion). The sub-ms activation time constant of *I*_Ca_ in whole-cell (τ_a_ = 0.77±0.08 ms at −21 mV, *n* = 6: Figure 3C, inset) was comparable to the sub-ms activation of the ensemble average. These results indicate that the macroscopic *I*_Ca_ recorded from IHCs of the middle cochlear region can be sufficiently well described by the summed behavior of the single Ca^2+^ channel. The macroscopic current-voltage (*I*-*V*) curve (Figure 3D) shows that *I*_Ca_ begins to activate positive to −60 mV. However, due to the surface charge screening effect in elevated extracellular Ca^2+^, this value is slightly (few mV) overestimated, i.e. less negative than expected in physiological (1.3 mM) extracellular Ca^2+^ concentration [31], [32]. Given an average peak *I*_Ca_ amplitude of 310 pA at −20 mV, with an elementary current extrapolated at the same potential of 0.33 pA (Figure 2C) and a *P*_o_ of 0.058, the total number of about Ca^2+^ channel per IHC is likely to be in the order of 16,000 (eqn. 1). Considering an average number of active presynaptic sites (active zones) per IHC of 21 (Figure 1), we calculated that there would be about 770 Ca^2+^ channels in each presynaptic active zone, which is about four times higher than that calculated in basal IHCs (∼180; [4]).

### Very short delay-to-first Ca^2+^ channel opening

**Figure 4:**
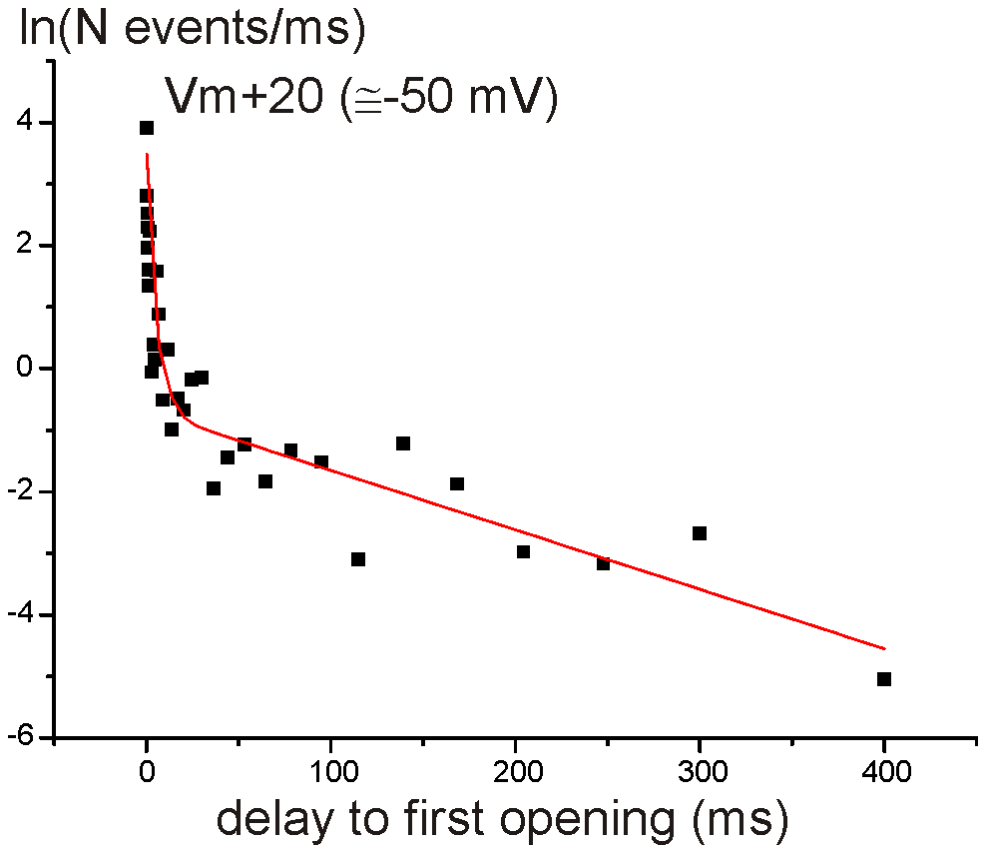
First latency of single Ca^2+^ channel opening. First latency distribution was obtained by plotting the natural logarithm of the number of observations ms^−1^ as a function of the delay between the stimulus (Vm+50) onset and the first observed Ca^2+^ channel opening at. The distribution was well defined by the sum of three exponentials (eqn. 2).

We further investigated the single Ca^2+^ channel activation kinetics by analyzing the first-latency, which is the delay between the stimulus onset and the first observed Ca^2+^ channel opening. The latency distribution obtained by plotting data from 6 patches could be well fitted by the sum of two (*V*_m_+20 mV; not shown) or three (*V*_m_+50 mV; Figure 4) exponentials. Time constant values and their relative weight are shown in Table 1. We found that the first latency decreased with membrane depolarization. Furthermore, at near −18 mV (*V*_m_+50 mV), but not at −50 mV (*V*_m_+20 mV), the latency showed a sub-ms time constant (0.15 ms). These results show that the average waiting time for an IHC Ca^2+^ channel to open decreases with depolarization, which is consistent with macroscopic I_Ca_ activation kinetics accelerating with depolarization [7].

**Table 1:** Time constants (*τ*) and the relative contributions (*W*, %) were obtained from the exponential fits of the latency of the first opening at two different membrane voltages.

	*τ*_1_ (ms)	*w_1_*	*τ*_2_ (ms)	*w_2_*	*τ*_3_ (ms)	*w_3_*
Vm+20 mV (∼−50 mV)	6.24	17	144	83	-	-
Vm+50 mV (∼−18 mV)	0.15	24	4.2	22	104	53

### Dwell time analysis reveals a complex gating behavior

**Figure 5:**
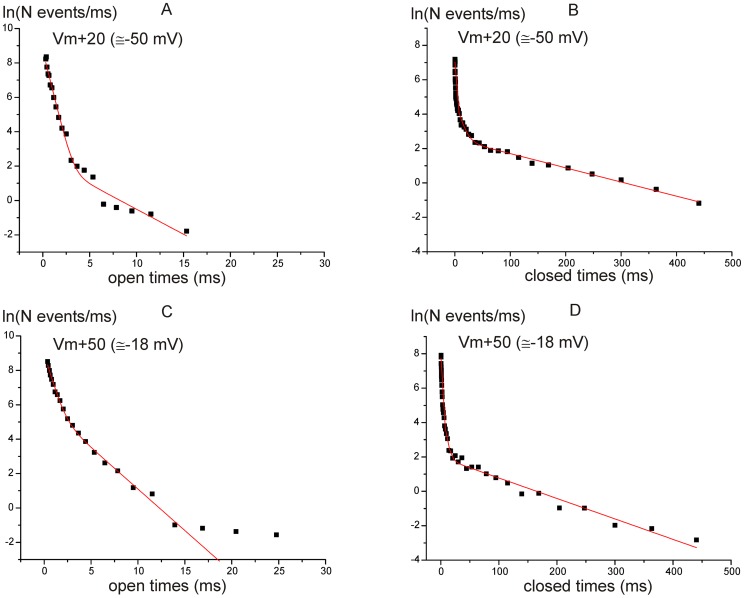
Kinetic properties of single Ca^2+^ channels. A–D, Semi-logarithmic plots of dwell times distributions at Vm+20 mV (A and B) and Vm+50 mV (C and D) in middle turn IHCs. Time intervals were binned at 16–20 bins/decade. Data were fitted by the sum of two or three exponential functions (eqn. 2). For the fitting shown in panel C, the last point, which appears to be representative of a third, very slow time constant that is however poorly resolved due to the small number of observations for such long open times, was ignored. The values for open (A and C) and closed (B and D) time constants with their relative weight and number of events are reported in Tables 2 and 3.

Next, we analyzed the distribution of the channel open and closed lifetimes at the two membrane depolarization levels (*V*_m_+20 mV and *V*_m_+50 mV; *n* = 6). Fitting the dwell time distributions with eqn. 2 (Figure 5) revealed two open (τ_o1_ and τ_o2_) and three closed (τ_c1_, τ_c2_ and τ_c3_) time constants (Tables 2 and 3, respectively). The values of these time constants could be grouped as follows: τ_1_ below 1 ms, τ_2_ between 1 ms and 10 ms, τ_3_ larger than 10 ms. The analysis of the distinct kinetic parameters revealed that the values of the open and closed time constants were similar between the two transmembrane voltages. However, there was an overall increase of the relative weight of τ_o2_ with depolarization. Moreover, the relative weight of the shortest close time constant (τ_c1_) increased with depolarization. This indicates that membrane depolarization caused an increased Ca^2+^ channel mean open time due to a higher number of openings, though with similar lifetime and kinetics. A similar result, i.e. depolarization changing the relative importance of dwell times constants but not their absolute values, was also found in immature apical mouse IHCs [18], and in adult basal gerbil IHCs [4], and thus it appears as a typical feature of IHC Ca^2+^ channels.

**Table 2:** Open time constants (*τ*_o_) and the relative contributions (*W*, %) were obtained from the exponential fits of the open time distributions at two different membrane voltages.

	*τ*_o1_ (ms)	*W_o1_*	*τ*_o2_ (ms)	*W_o2_*	*τ*_o3_ (ms)	*W_o3_*	n° of events
Vm+20 mV (∼−50 mV)	0.46	97	3.4	4			2693
Vm+50 mV (∼−18 mV)	0.52	81	2.1	19			3887

**Table 3:** Closed time constants (*τ*_c_) and the relative contributions (*W*, %) were obtained from the exponential fits of the closed time distributions at two different membrane voltages.

	*τ*_c1_ (ms)	*W_c1_*	*τ*_c2_ (ms)	*W_c2_*	*τ*_c3_ (ms)	*W_c3_*	n° of events
Vm+20 mV (∼−50 mV)	0.45	23	8.1	30	121	47	3097
Vm+50 mV (∼−18 mV)	0.68	62	4.1	23	84	15	3594

### Ca^2+^ channels preferentially open in bursts

**Figure 6:**
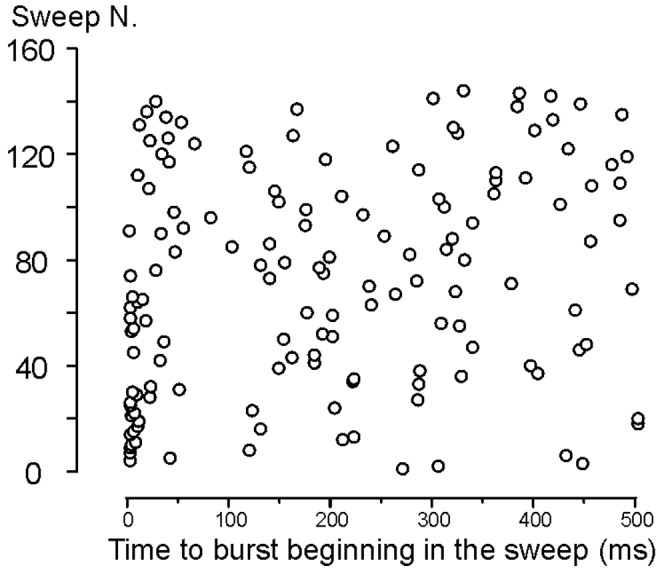
Single Ca^2+^ channels open in bursts. Time of burst beginning in the sweeps recorded at Vm+50 mV for middle turn IHCs. Note that bursts appear more frequently at the very beginning of the sweep.

Analysis of the closed-time distribution (Figure 5D; Table 3) revealed the presence of a very slow exponential component with a mean time constant (τ_c3_) of 84 ms (Vm+50 mV). τ_c3_ was about 20 times greater than the “intermediate” time constant of the closed-time distribution (τ_c2_: 4.1 ms). Moreover, the relative weight of the slowest exponential component was only 15% compared to the total. Therefore, the average number of “long” closures per sweep was exceeded by that of “short” closures, and single Ca^2+^ channel openings had a relatively high probability of being separated from each other by short closings. This implies that Ca^2+^ channel activity was largely organized in bursts, consisting of clusters of openings separated by short closings, and interrupted by prolonged closures (see Figure 2A and 3A). In order to analyze the properties of burst openings, we first defined a burst as any cluster of openings occurring without superimpositions and separated by the previous and/or following openings by an interval of at least 8 ms (i.e. twice the value observed for τ_c2_). The mean duration of the burst (for bursts not terminated by the end of the sweep) was 19±20 ms (*n* = 137 from 6 patches). Burst onsets were concentrated at the very beginning of the sweep (Figure 6), consistent with the short Ca^2+^ channel first latency.

### Ca^2+^ channels deactivate quickly upon repolarization

**Figure 7:**
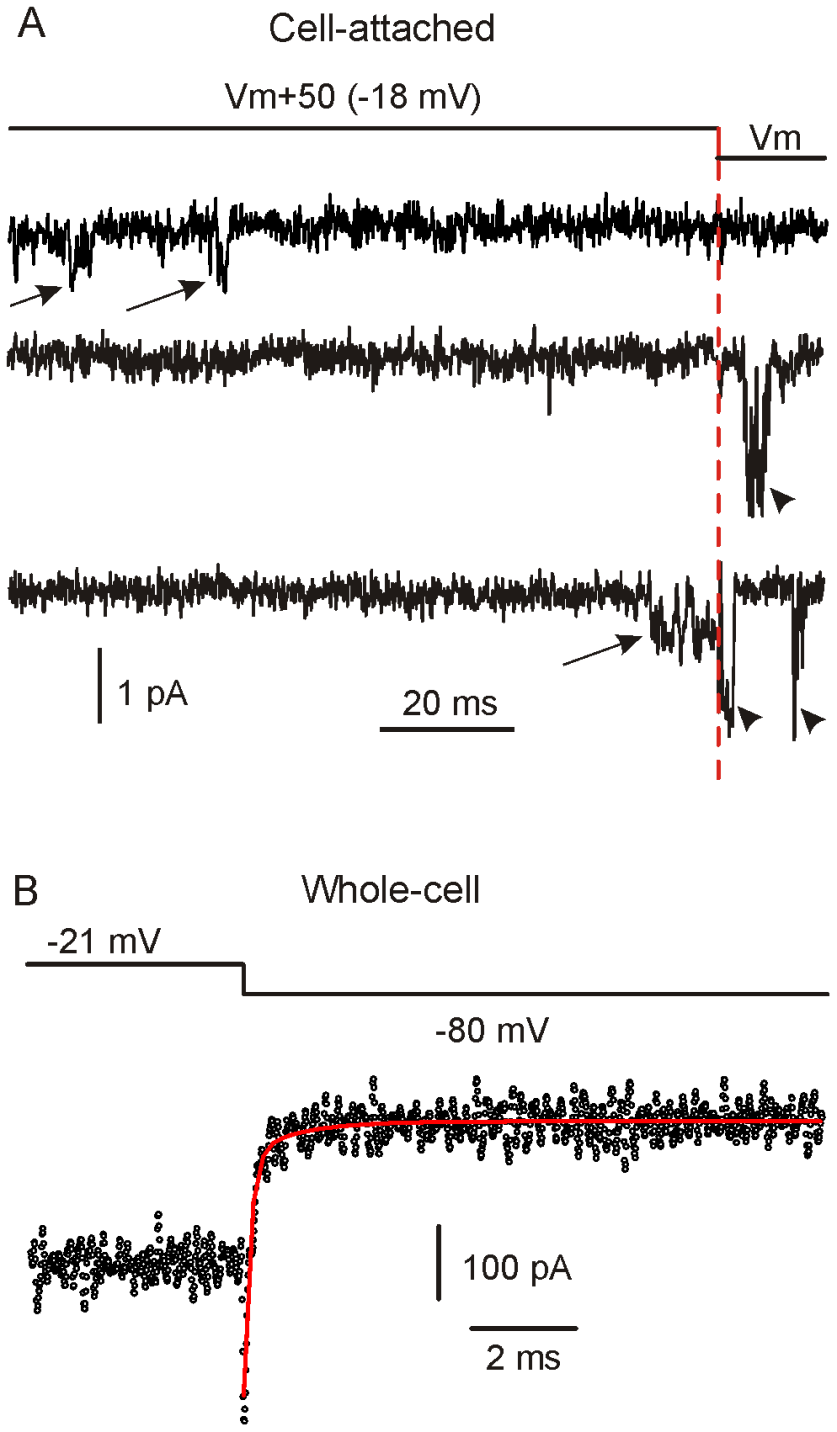
Ca^2+^ channel activity upon repolarization. A, The first portion of the trace shows Ca^2+^ channel activity at *V*_m_+50 (arrows), while the last portion, as also indicated by the red vertical dashed line, shows Ca^2+^ channel activity upon repolarization to *V*_m_ (arrowheads). Like the trace shown at the top, the majority of the traces showing Ca^2+^ channel openings at *V*_m_+50 did not show any openings upon repolarization. The second and third traces from the top are rare examples of Ca^2+^ channel activity upon repolarization. Note the increase in current amplitude upon repolarization due to the increased driving force. In the bottom trace, a Ca^2+^ channel was open at *V*_m_+50 mV just before repolarization. **B**, Macroscopic tail current elicited by repolarizing the cell from −21 mV to −80 mV. In order to increase the signal-to-noise ratio, the fit was performed on the average current recorded from six IHCs. Note the fast time course of *I*_Ca_ deactivation, which was fitted (red line) by a double exponential function.

We found that upon IHC membrane repolarization, the majority (∼90%; 173/193) of the sweeps did not show any channel activity (Figure 7A, top trace). Out of the small number of sweeps showing some Ca^2+^ channel activity during the repolarization step, a few showed an occasional opening after a “silent” period (Figure 7A; middle trace), while the majority represented the instantaneous current through a channel already open just before repolarization (Figure 7A, bottom trace). The almost absent Ca^2+^ channel activity upon repolarization, is consistent with the rapid deactivation of the macroscopic *I*_Ca_ (Figure 7B). Indeed, *I*_Ca_ deactivation, elicited by a repolarizing step from −21 mV to −80 mV, was extremely rapid and best fitted with a double exponential function (τ_fast_ = 0.13 ms; τ_slow_ = 1.13 ms, with τ_fast_ contribution being seven fold larger than τ_slow_). Thus, the deactivation kinetics of *I*_Ca_ is dominated by a very fast component, with the slower one likely resulting from BayK 8644 [21].

## Discussion

In the present study we have characterized the biophysical properties of voltage-gated Ca^2+^ channels in adult gerbil IHCs of the middle cochlear region (low frequency: ∼2 kHz). We found that most of the macroscopic and elementary properties of the Ca^2+^ current, namely the sub-ms activation and deactivation kinetics, the slow inactivation, the elementary conductance and the bursting activity, closely resemble those previously described in basal-region IHCs (high frequency: ∼30 kHz: [4]). This similarity is consistent with a predominant expression of the Ca_V_1.3 Ca^2+^ channel α-subunit in the mammalian cochlear hair cells [1], [5]. However, Ca^2+^ channels present in IHCs of the middle cochlear region showed on average a much lower *P*_o_ and shorter burst duration in response to sustained depolarization than those reported for basal cells [4]. The elementary Ca^2+^ channel properties in IHCs of the middle cochlear region appear to be best suited to follow the a.c. component of their receptor potential and thus phase-lock cell's neurotransmission to low-frequency sounds.

### Mid-cochlear turn IHC Ca_V_1.3 channels show very rapid activation and deactivation

We found that the fastest time constant of Ca^2+^ channel first latency (τ_1_) was ∼0.15 ms (at −18 mV, 5 mM Ca^2+^ and 34–37°C). This value is similar to that found in adult basal IHCs in analogous experimental conditions (∼0.18 ms), which is assumed to decrease to about 40 µs in the presence of 1.3 mM instead of 5 mM Ca^2+^ [4] because of surface screening effects [31], [32], [33]. A similar rapid (sub-ms) activation kinetics was also recorded from the macroscopic Ca^2+^ current. We have also found that Ca^2+^ channel openings were rarely seen upon repolarization following depolarizing steps, which is consistent with very rapid macroscopic tail currents (Figure 7). L-type Ca^2+^ channels have been reported to show enhanced Ca^2+^ channel activity early after repolarization from positive voltage levels and generate slow tails currents [34], though the Ca_V_1 isoform/s involved were not identified. In many tissues Ca_V_1.3 channels are expressed together with other Ca_V_1 isoforms [28], whereas in IHCs *I*_Ca_ is carried almost exclusively by Ca_V_1.3 (>90%: [1]). Therefore, it is reasonable to assume that the rapid (sub-ms) activation and deactivation kinetics observed here is a specific property of the Ca_V_1.3 Ca^2+^ channel isoform, which is consistent with a previous report on chicken cochlear hair cells [35], mouse outer hair cells [36] and in heterologous expression systems [37].

### Depolarization favors long openings

Analysis of dwell time distributions revealed a complex gating behavior that is similar to that found in basal IHCs [4], with two open and three closed time constants (Table 2 and 3). With depolarization the relative weight of long openings and short closures increased, which is consistent with the increase in size of the macroscopic *I*_Ca_ with depolarization. The absolute τ values were also similar to those found in basal IHCs [4], although their relative weight at comparable membrane potentials indicates a higher percentage of long duration openings in basal than mid-cochlear turn IHCs, which is consistent with the overall larger *P*_o_ in basal cells.

### Depolarization induces the earlier appearance of brief bursts of Ca^2+^ channel activity

Dwell time analysis also indicates that single Ca^2+^ channel openings had a relatively high probability of being separated by short closings despite the very low *P*_o_. This implies that Ca^2+^ channel activity was largely organized in bursts. As also found in basal IHCs [4], burst activity was most frequent during the initial part of the depolarizing response (Figure 6). When we limited the analysis of *P*_o_ to the first 40 ms of each sweep, indeed, its value increased to 0.058, i.e. ∼ twice the 500 ms value. A decrease in *P*_o_ during the 500 ms step was consistent with partial inactivation of the ensemble average current (Figure 3C) and of the macroscopic current (Figure 3D).

### Diversity of elementary Ca^2+^ channel properties

The elementary properties of voltage-dependent Ca^2+^ channels have been characterized in vestibular and cochlear hair cells of lower vertebrates and mammals [4], [18], [36], [37], [38]. Synaptic vesicle fusion at IHC presynaptic active zones is controlled almost exclusively by Ca^2+^ entry through L-type (Ca_V_1.3) Ca^2+^ channels [2], [5], which show a negative voltage activation range [4], [18]. These channels show only partial inactivation and a complex gating, characterized by multiple open and closed time constants, together with periods of no activity. The elementary Ca^2+^ current activation kinetics (delay-to-first opening) were found to be slower in immature [18] compared to mature IHCs ([4]; present study).

Recently, it has been shown that Ca_V_1.3 channels expressed in the same IHC can be functionally heterogeneous [19], [39], possibly as a consequence of different intracellular modulation, alternative alpha1-subunit splicing or subunit composition (e.g. association with different beta-subunit isoforms or splice-variants) [40], [41]. In principle, Ca_V_1.3 channels could vary in several elementary properties, e.g. conductance, voltage- or Ca^2+^-sensitivity, kinetics, etc. The most obvious difference that we found in Ca_V_1.3 channel properties between IHCs from the basal [4] and middle cochlear turn was the lower *P*_o_ of the latter. Given the overall low frequency of Ca^2+^ channel openings, the *P*_o_ value was largely determined by the channel entering the bursting modality of opening. Consistent with this observation, mean burst duration was on average shorter in mid-cochlear IHCs (19 ms) compared to that of basal cells (81 ms: [4]), the latter showing a larger *P*_o_ than the former. We found that bursting activity appeared clustered in successive sweeps, as previously found in recordings from basal IHCs [4], and more generally for L-Type Ca^2+^ channels [21]. This phenomenon has been attributed to some cellular modulation or metabolic state [23], [29], [30]. This suggests that functionally different Ca_V_1.3 channels among IHCs, and possibly between presynaptic active zones in a same IHC, are achieved by modulating the channel bursting modality.

### Number of Ca^2+^ channels per IHC

The total number of Ca^2+^ channels per IHC (about 16,000) was estimated using eqn. 1, which uses values obtained under different experimental conditions (whole-cell and cell-attached recordings) that could have caused an overestimation. One crucial factor required to maintain Ca_V_1.3 channel activity is the concentration of intracellular ATP [42], which may vary between whole-cell and cell-attached configurations. While whole-cell recordings were performed using 5 mM ATP, the concentration of the cytosolic ATP in the unperturbed IHC (cell-attached recordings) is not known, but it could be lower [42], [43]. Moreover, the possible fine intracellular modulation of Ca^2+^ channels [44] is likely to be lost in whole-cell recordings. Therefore the Ca^2+^ channel *P*_o_ in whole-cell recordings could be artifactually augmented by the experimental condition, resulting in an overestimation of the Ca^2+^ number per IHC. Despite this possibility, lower-frequency middle-coil IHCs (about 300 Hz) express about 4-times more Ca^2+^ channels compared to high-frequency basal cells ([4]: about 30 kHz), under the same experimental conditions. This larger Ca^2+^ channel number in middle-turn IHCs is likely to increase the dynamic range for exocytosis in low-frequency cells showing a phasic (a.c.) component, revealing a major difference among cells along the mammalian cochlea.

### Functional significance of Ca^2+^ channel properties in IHCs of the gerbil mid-cochlear turn

IHC properties change along the length of the gerbil cochlea [45] in order to process sound at their characteristic frequency, which varies from about 0.1 to 60 kHz [46]. It has been suggested that the properties of one or very few Ca^2+^ channels are likely to govern the fusion of each docked vesicle at IHC ribbon synapses (nanodomain control of exocytosis: [3], [47], [48]. Therefore, knowledge of elementary Ca^2+^ channel properties, together with their functional coupling with vesicles, is an essential pre-requisite to understand how IHC ribbons release neurotransmitter with the required precision of timing. We found that Ca^2+^ channels in middle-coil IHCs promptly (sub-ms latency) respond to depolarization with short bursts of activity and to repolarization with very fast deactivation. This would ensure phase-locked Ca^2+^ influx in response to sinusoidal acoustic stimuli up to a few kHz [11]. Similar, fast activation kinetics were also found for basal IHCs Ca^2+^ channels [4]. Although basal IHCs are not expected to be able to follow their characteristic high frequency stimuli, the rapid activation/deactivation kinetics would enable them to signal the beginning and the end of a high-frequency sound with just minimal delays, which is important for binaural sound localization [49], [50].

As previously found for basal IHCs, the Ca^2+^ channel *P*_o_ was largely determined by the bursting mode of gating, and was otherwise (i.e. outside bursts) negligible. This property is even more prominent in mid-cochlear IHCs, and together with the relatively high prevalence of null sweeps (55%), indicates that upon depolarization only a small fraction of the Ca^2+^ channels at each active site will open. This suggests a “volley principle” for *I*_Ca_-coupled neurotransmitter exocytosis such that by increasing sound intensity it will increase the probability that a vesicle fusion event will occur at each active site. This might explain how increasing sound intensity can be encoded at the single afferent auditory nerve axons as an increase in the number of cycles of the sound wave that elicit a phase-locked action potential [51].

With depolarization, the macroscopic I_Ca_ increases and accelerates, consistent with single channel *P*_o_ increasing and latency to first opening decreasing. This would ordinarily be expected to reduce synaptic delay as at other chemical synapses [52], [53], thus producing a phase advance with increasing stimulus intensity. Indeed, synaptic delay was shown to decrease with increasing depolarization for hair cells that are initially at rest [54], [55]. However, the relative timing of afferent fibre action potentials remains constant throughout their intensity range [12], [54], [56]. It has been proposed that Ca^2+^ channel facilitation at membrane voltages close to rest [54], [57] and vesicle pool depletion at more depolarized voltages balance each other, so that for sine-wave stimuli of different intensities the average phase will be conserved [54]. Also, it has recently been reported that multivesicular release, which requires Ca^2+^ inflow but does not appear to depend upon the amplitude of Ca^2+^ inflow [47], could be responsible for EPSC phase-lock at all stimulus intensity [55]. Here, we infer that during the repolarizing phase of each cycle of the acoustic stimuli, the single-channel Ca^2+^ current will likely reaches its maximal amplitude because of the increased driving force (the elementary “tail” current in Figure 7A, middle and bottom traces). Since only ∼4% of the IHC mechano-transducer (MET) channels are open at rest [58], the IHC depolarizing peak produced by the excitatory phase of the sound wave can vary largely depending on the sound intensity (louder sounds: more MET channels open; dynamic range >90% of the MET channels), while the hyperpolarizing peak will vary little. Thus, the amplitude of the elementary tail current will result solely dependent to the (invariable) Ca^2+^ equilibrium potential, and not to the stimulus intensity, and we propose that this brief maximal elementary Ca^2+^ signal is able to evoke a (multivesicular?) release event which, because of the very fast deactivation kinetics of Ca_V_1.3 channels, would occur in a phase-locked manner while minimizing asynchronous vesicle release (see also ref. [55]). This Ca^2+^ signal will occur with a probability that will reliably reflect the open-channel probability increase during the foregoing depolarization and, hence, the stimulus intensity. In other words, the chance to see an elementary tail current will be greater the larger the previous depolarization, in keeping with the above hypothesized volley principle.

In summary, the elementary properties of Ca^2+^ channels expressed in mid-cochlear IHCs appear to show specific biophysical properties that make these channels ideal to code the timing and the intensity of the sound wave in the frequency range to which gerbils are most sensitive [16].

## Materials and Methods

### Ethics Statement

All animal work has been conducted according to relevant national and international guidelines. All gerbils of either sex were killed by cervical dislocation, under Schedule 1 in accordance with UK Home Office regulations. Our animal work adhere to the NC3Rs guideline (ARRIVE). Specifically, the protocol was approved by: 1) In Italy animal studies were licensed by the Ministero dell'Istruzione, Università e Ricerca, Rome, and approved by the Committee on the Ethics of Animal Experiments of the University of Pavia; 2) In the UK, all animal studies were licensed by the Home Office under the Animals (Scientific Procedures) Act 1986 and were approved by the University of Sheffield Ethical Review Committee; 3) In Germany, care and use of the animals and the experimental protocol were reviewed and approved by the animal welfare commissioner and the regional board for scientific animal experiments in Tubingen. A total number of 41 adult (from postnatal day 20 (P20) to P37, where the day of birth is P0) gerbils were sacrificed, of which 25 animals provided useful data as follows: 3 for immunocytochemistry, 8 for single-channel recordings in high-K^+^ extracellular solution, 8 for single-channel recordings in high-Na^+^ extracellular solution, and 6 for whole-cell recordings.

### Patch-clamp recording

Inner hair cells (IHCs) from gerbils were studied in acutely dissected organs of Corti. Recordings were obtained from IHCs positioned in the middle turn of the adult cochlea corresponding *in vivo* to mean characteristic frequencies of ∼2 kHz [59]. The organs of Corti were dissected as previously described [43], [60] in normal extracellular solution (in mM): 135 NaCl, 5.8 KCl, 1.3 CaCl_2_, 0.9 MgCl_2_, 0.7 NaH_2_PO_4_, 5.6 D-glucose, 10 Hepes-NaOH, 2 sodium pyruvate, amino acids and vitamins (pH 7.5; osmolality ∼308 mmol kg^−1^). Cochleae were viewed using an upright microscope (Leica DMLFS, Germany). Unless otherwise stated, all recordings were performed near body temperature (35–37°C) and with the normal extracellular solution as the bath solution.

For single Ca^2+^ channel recordings, patch pipettes were made from quartz glass capillaries (Sutter Instruments, USA) coated with surf wax (Mr Zoggs SexWax, USA) to minimise the fast electrode capacitative transient. Patch pipettes contained the following solution (in mM): 5 CaCl_2_, 102 CsCl, 10 Hepes-KOH, 15 4-AP and 40 TEA (pH 7.5). Linopirdine (100 µM: Tocris, Bristol, UK), niflumic acid (50 µM: Sigma, UK) and BayK 8644 (5 µM: Sigma) were added to the pipette solution. Stock solutions of niflumic acid and BayK 8644 were prepared in DMSO and stored at −20°C (final dilution 1∶2000). In a few experiments (Figure 2A,B) the membrane potential of IHCs was zeroed by superfusing a high-K^+^ extracellular solution [18] containing (in mM): 140 KCl, 0.2 CaCl_2_, 6.2 MgCl_2_, 0.7 NaH_2_PO_4_, 5.6 D-glucose, 15 Hepes-KOH (pH = 7.5) In some initial experiments trypsin (0.025–0.05% v/v) was very briefly and topically applied onto IHCs prior attempting to seal. Data were filtered at 2 or 5 kHz (4-pole Bessel) and sampled at 20 or 50 kHz. In very few cases, current traces were additionally filtered offline at 1 kHz (8-pole Bessel). Membrane potentials were corrected for the liquid junction potential of +3 mV.

Whole-cell recordings were performed using soda glass capillaries (resistance 2–3 MΩ) coated with surf-wax and filled with (in mM): 106 Cs-glutamate, 20 CsCl, 3 MgCl_2_, 1 EGTA-CsOH, 5 Na_2_ATP, 0.3 Na_2_GTP, 5 Hepes-CsOH, 10 Na_2_-phosphocreatine (pH 7.3). Inward Ca^2+^ currents were recorded in isolation by superfusing IHCs with a high_Na^+^ extracellular solution (in mM): 103 NaCl, 5.8 KCl, 5 CaCl_2_, 0.9 MgCl_2_, 0.7 NaH_2_PO_4_, 5.6 D-glucose, 10 Hepes-NaOH, 30 TEACl, 15 4-AP (pH 7.5; osmolality ∼306 mmol kg^−1^) daily added with 100 µM linopirdine, 50 µM niflumic acid and 5 µM BayK 8644. Recordings were filtered at 5 or 10 kHz (8-pole Bessel) and sampled at 50 or 100 kHz. Membrane potentials were corrected for residual series resistance (*R*_s_: 1.27±0.05 MΩ, *n* = 6) and liquid junction potential (−11 mV). Leakage and residual capacitative transients were subtracted off-line by scaling the current and artifacts generated by a voltage step from −80 mV to −70 mV, a voltage range at which the contribution by voltage- and time-dependent currents is negligible or absent in the presence of a Cs^+^-based intracellular solution.

### Immunocytochemistry

Cochleae from adult gerbil (P20) were used to prepare cryosections for immunofluorescence microscopy and processed as previously described [61]. Briefly, cochleae were dissected and fixed for 2 hrs with 2% paraformaldehyde (w/v), decalcified, embedded in Tissue-Tek optimal cutting temperature compound (Sakura Finetek) and cryosectioned at a thickness of 10 µm. Sections were embedded with Vectashield mounting medium with DAPI (Vector Laboratories). Antibodies directed against Ca_V_1.3 (rabbit, Alomone Laboratories, diluted 1∶50) and Ribeye/CtBP2 (mouse, BD Transduction Laboratories, diluted 1∶50) were used for cryosection preparations. Primary antibodies were detected with Cy3-conjugated (Jackson ImmunoResearch Laboratories) or Alexa Fluor 488–conjugated (Molecular Probes) secondary antibodies. Sections were viewed using an Olympus BX61 microscope equipped with motorized z-axis, epifluorescence illumination and differential interference contrast (DIC). Images were acquired using a CCD camera and analyzed with cellSense Dimension software (OSIS GmbH, Münster, Germany). To display Ca^2+^ channel and ribbon distribution, cochlea slices were imaged over a distance of several µm with the coverage of the IHC synaptic region in an image-stack along the z-axis (z-stack) followed by three-dimensional deconvolution using cellSense Dimension module with the advanced maximum likelyhood estimation algorithm (ADVMLE, OSIS). The immuno- histological figures display composite images, which represent the maximum intensity projection over all layers of the z-stack. Immunopositive spot counting and co-localization was performed as previously described [18]. Immunolabeling has been done on three animals as follows: one cochlea, four different counts; one cochlea, four different counts; both cochleae, three counts + two counts, for a total of 13 IHC counts.

### Data analysis

Single Ca^2+^ channel analysis was performed as previously described [18] using Clampfit (Molecular Devices) and Origin (OriginLab, USA). Briefly, leak and uncompensated capacitive currents were corrected by subtracting average episodes without channel activity (null sweeps) from the active sweeps. Event detection was performed with the 50% threshold detection method with each transition visually inspected before being accepted. Idealized traces were used to calculate single channel amplitude distribution (event duration >0.34 ms), open probability (*P*_o_) and open and closed time histograms. *P*_o_ was calculated as the time fraction spent in the open time vs. the total recording time [18]. Dwell times distributions were fitted with multiple exponentials.

The total number of Ca^2+^ channels expressed in IHCs was estimated using the following equation: 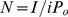
(eqn.1)where *N* is the total number of channels, *I* is the size of the macroscopic Ca^2+^ current measured using 500 ms voltage steps, *i* is the single-channel current size and *P*_o_ the open channel probability.

The distributions of open and closed times were analyzed using log–log plots [62]. Dwell-time lower and upper bin limits were first set according to a logarithmic scale (12 bins per decade). After binning, the number of events (*n*) was divided by the corresponding bin width (*δt_i_*), and the natural logarithm of *n_i_*/*δt_i_* ratio was calculated. These values were plotted as a function of *x*  =  ln*t* to construct log–log frequency distribution graphs. Exponential fitting of log–log histograms was performed by applying them following double-logarithmic transform of a sum of exponential equations [62]:
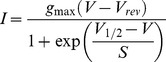
(eqn.2)where *x*_0*j*_  =  ln*τ_j_*, and *W_j_* and *τ_j_* are the weight coefficient and time constant, respectively, for each exponential component. The plots and fittings obtained in this way were then shown on a linear time scale for better clarity (see Figs. 4 and 5).

The first latency distribution was investigated by measuring the time interval between the first point of the capacitative transient and the first Ca^2+^ channel opening. These values were corrected for the number of channels in the patch [63]. The number of events used for this analysis was smaller than those used for the dwell times, since only the time to the first opening from each trace could be used. The distribution of the first latency was analysed as for the open and closed times. In a few sweeps Ca^2+^ channel were already open at the onset of the voltage step. These “zero delay” openings were not included in the analysis because obtained while *V*_m_ was varying.The macroscopic current-voltage curve was fitted with the following equation:
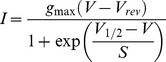
(eqn.3)where *I* is the current, *g*_max_ is the maximum chord conductance, *V* is the membrane potential, *V*_rev_ is the reversal potential of the current, *V*_½_ is the potential at which the conductance is half activated and *S* is the slope factor that defines the voltage sensitivity of current activation. In the text and figures, mean values are quoted ± SEM unless otherwise specified. To determine if the mean *P*_o_ obtained from each sweep during the first 40 ms or the for its whole duration (500 ms) were significantly different, F-test followed by *t*-test were used. In the text, *n* refers to the number of cell/patches/sweeps. Mean values are provided ± S.D.

## References

[1] Platzer J, Engel J, Schrott-Fischer A, Stephan K, Bova S, et al (2000) Congenital deafness and sinoatrial node dysfunction in mice lacking class D L-type Ca^2+^ channels. Cell 102: 89–97. 10929716 10.1016/s0092-8674(00)00013-1

[2] Kim MH, Li GL, von Gersdorff H (2013) Single Ca^2+^ channels and exocytosis at sensory synapses. J Physiol 591(13): 3167–3178. 23459757 10.1113/jphysiol.2012.249482 PMC3717220

[3] Brandt A, Khimich D, Moser T (2005) Few Ca_V_1.3 channels regulate the exocytosis of a synaptic vesicle at the hair cell ribbon synapse. J Neurosci 25: 11577–11585. 16354915 10.1523/JNEUROSCI.3411-05.2005 PMC6726013

[4] Zampini V, Franz C, Magistretti J, Johnson SL, Knipper M, et al (2013) Burst activity and ultrafast activation kinetics of Ca_V_1.3 Ca^2+^ channels support presynaptic activity in adult gerbil hair cell ribbon synapses. J Physiol 591: 3811–3820. 23713031 10.1113/jphysiol.2013.251272 PMC3764630

[5] Brandt A, Striessnig J, Moser T (2003) Ca_V_1.3 channels are essential for development and presynaptic activity of cochlear inner hair cells. J Neurosci 23: 10832–10840. 14645476 10.1523/JNEUROSCI.23-34-10832.2003 PMC6740966

[6] Johnson SL, Marcotti W, Kros CJ (2005) Increase in efficiency and reduction in Ca^2+^ dependence of exocytosis during development of mouse inner hair cells. J Physiol 563(1): 177–191. 15613377 10.1113/jphysiol.2004.074740 PMC1665557

[7] Johnson SL, Marcotti W (2008) Biophysical properties of Ca_V_1.3 calcium channels in gerbil inner hair cells. J Physiol 586(4): 1029–1042. 18174213 10.1113/jphysiol.2007.145219 PMC2268984

[8] Glowatzki E, Fuchs PA (2002) Transmitter release at the hair cell ribbon synapse. Nat Neurosci 5: 147–154. 11802170 10.1038/nn796

[9] Liberman MC (1980) Morphological differences among radial afferent fibers in the cat cochlea: an electron-microscopic study of serial sections. Hear Res 3: 45–63. 7400048 10.1016/0378-5955(80)90007-6

[10] Sterling P, Matthews G (2005) Structure and function of ribbon synapses. Trends Neurosci 28: 20–29. 15626493 10.1016/j.tins.2004.11.009

[11] Palmer AR, Russell IJ (1986) Phase-locking in the cochlear nerve of the guinea-pig and its relation to the receptor potential of inner hair-cells. Hear Res 24: 1–15. 3759671 10.1016/0378-5955(86)90002-x

[12] Fuchs PA (2005) Time and intensity coding at the hair cell's ribbon synapse. J Physiol 566: 7–12. 15845587 10.1113/jphysiol.2004.082214 PMC1464726

[13] Pickles JO (1996) An Introduction to the Physiology of Hearing. Academic Press Inc., San Diego.

[14] Rossing TD (2007) Springer Handbook of Acoustics. Springer, New York.

[15] Dallos P (1985) Response characteristics of mammalian cochlear hair cells. J Neurosci 5: 1591–1608. 4009248 10.1523/JNEUROSCI.05-06-01591.1985 PMC6565270

[16] Ryan A (1976) Hearing sensitivity of the mongolian gerbil, *Meriones unguiculatus* . J Acoust Soc Am 59: 1222–1226. 956517 10.1121/1.380961

[17] Ehret G (1975) Masked auditory thresholds, critical ratios, and scales of the basilar membrane of the house mouse (*Mus musculus*). J Comp Physiol 103: 329–341.

[18] Zampini V, Johnson SL, Lawrence N, Franz C, Münkner S, et al (2010) Elementary properties of Ca_V_1.3 Ca^2+^ channels expressed in mouse inner hair cells. J Physiol 588: 187–199. 19917569 10.1113/jphysiol.2009.181917 PMC2817446

[19] Meyer AC, Frank T, Khimich D, Hoch G, Riedel D, et al (2009) Tuning of synapse number, structure and function in the cochlea. Nat Neurosci 12: 444–453. 19270686 10.1038/nn.2293

[20] Ceña V, Stutzin A, Rojas E (1989) Effects of calcium and Bay K-8644 on calcium currents in adrenal medullary chromaffin cells. J Membr Biol 112: 255–265. 2482362 10.1007/BF01870956

[21] Hess P, Lansman JB, Tsien RW (1984) Different modes of Ca^2+^ channel gating behaviour favoured by dihydropyridine Ca^2+^ agonists and antagonists. Nature 311: 538–544. 6207437 10.1038/311538a0

[22] Markwardt F, Nilius B (1988) Modulation of calcium channel currents in guinea-pig single ventricular heart cells by the dihydropyridine Bay K 8644. J Physiol 399: 559–575. 2457095 10.1113/jphysiol.1988.sp017096 PMC1191680

[23] Nowycky MC, Fox AP, Tsien RW (1985) Long-opening mode of gating of neuronal calcium channels and its promotion by the dihydropyridine calcium agonist Bay K 8644. Proc Natl Acad Sci USA 82: 2178–2182. 2580308 10.1073/pnas.82.7.2178 PMC397516

[24] Koschak A, Reimer D, Huber I, Grabner M, Glossmann H, et al (2001) alpha 1D (Ca_v_1.3) subunits can form l-type Ca^2+^ channels activating at negative voltages. J Biol Chem 276(25): 22100–22106. 11285265 10.1074/jbc.M101469200

[25] Safa P, Boulter J, Hales TG (2001) Functional properties of Ca_v_1.3 (alpha1D) L-type Ca^2+^ channel splice variants expressed by rat brain and neuroendocrine GH3 cells. J Biol Chem 276(42): 38727–38737. 11514547 10.1074/jbc.M103724200

[26] Scholze A, Plant TD, Dolphin AC, Nürnberg B (2001) Functional expression and characterization of a voltage-gated Ca_V_1.3 (alpha1D) calcium channel subunit from an insulin-secreting cell line. Mol Endocrinol 15(7): 1211–1221. 11435619 10.1210/mend.15.7.0666

[27] Xu W, Lipscombe D (2001) Neuronal Ca(V)1.3alpha(1) L-type channels activate at relatively hyperpolarized membrane potentials and are incompletely inhibited by dihydropyridines. J Neurosci 21(16): 5944–5951. 11487617 10.1523/JNEUROSCI.21-16-05944.2001 PMC6763157

[28] Lipscombe D, Helton TD, Xu W (2004) L-type calcium channels: the low down. J Neurophysiol 92: 2633–41. 15486420 10.1152/jn.00486.2004

[29] Carabelli V, Hernandez-Guijo JM, Baldelli P, Carbone E (2001) Direct autocrine inhibition and cAMP-dependent potentiation of single L-type Ca^2+^ channels in bovine chromaffin cells. J Physiol 532: 73–90. 11283226 10.1111/j.1469-7793.2001.0073g.x PMC2278521

[30] Kamp TJ, Hell JW (2000) Regulation of cardiac L-type calcium channels by protein kinase A and protein kinase C. Circ Res. 87: 1095–1102. 10.1161/01.res.87.12.1095 11110765

[31] Byerly L, Chase PB, Stimers JR (1985) Permeation and interaction of divalent cations in calcium channels of snail neurons. J Gen Physiol 85: 491–518. 2409216 10.1085/jgp.85.4.491 PMC2215805

[32] Hille B (2001) Ion Channels of Excitable Membranes. 3^rd^ edn. Sinauer Associates, Inc. Sunderland.

[33] Smith PA, Aschroft FM, Fewtrell CM (1993) Permeation and gating properties of the L-type calcium channel in mouse pancreatic beta cells. J Gen Physiol 101: 767–797. 7687645 10.1085/jgp.101.5.767 PMC2216780

[34] Thibault O, Porter NM, Landfield PW (1993) Low Ba^2+^ and Ca^2+^ induce a sustained high probability of repolarization openings of L-type Ca^2+^ channels in hippocampal neurons: physiological implications Proc Natl Acad Sci U S A. 90: 11792–11796. 10.1073/pnas.90.24.11792 PMC48070 7505447

[35] Zidanic M, Fuchs PA (1995) Kinetic analysis of barium currents in chick cochlear hair cells. Biophys J 68: 1323–1336. 7787021 10.1016/S0006-3495(95)80305-X PMC1282027

[36] Rodriguez-Contreras A1, Yamoah EN (2001) Direct measurement of single-channel Ca(2+) currents in bullfrog hair cells reveals two distinct channel subtypes. J Physiol 534(3): 669–689. 11483699 10.1111/j.1469-7793.2001.00669.x PMC2278743

[37] Rodríguez-Contreras A, Yamoah EN (2003) Effects of permeant ion concentrations on the gating of L-type Ca^2+^ channels in hair cells. Biophys J 84(5): 3457–3469. 12719271 10.1016/S0006-3495(03)70066-6 PMC1302902

[38] Zampini V, Valli P, Zucca G, Masetto S (2006) Single-channel L-type Ca^2+^ currents in chicken embryo semicircular canal type I and type II hair cells. J Neurophysiol 96(2): 602–612. 16687612 10.1152/jn.01315.2005

[39] Frank T, Khimich D, Neef A, Moser T (2009) Mechanisms contributing to synaptic Ca^2+^ signals and their heterogeneity in hair cells. Proc Natl Acad Sci USA 106(11): 4483–4488. 19246382 10.1073/pnas.0813213106 PMC2657422

[40] Bock G, Gebhart M, Scharinger A, Jangsangthong W, Busquet P, et al (2011) Functional properties of a newly identified C-terminal splice variant of Cav1.3 L-type Ca^2+^ channels. J Biol Chem 286: 42736–42748. 21998310 10.1074/jbc.M111.269951 PMC3234942

[41] Singh A, Gebhart M, Fritsch R, Sinnegger-Brauns MJ, Poggiani C, et al (2008) Modulation of voltage- and Ca^2+^-dependent gating of Ca_V_1.3 L-type calcium channels by alternative splicing of a C-terminal regulatory domain. J Biol Chem 283: 20733–20744. 18482979 10.1074/jbc.M802254200 PMC2475692

[42] Weiler S, Krinner S, Wong AB, Moser T, Pangršič TJ (2014) ATP hydrolysis is critically required for function of Ca_V_1.3 channels in cochlear inner hair cells via fueling Ca^2+^ clearance. J Neurosci 34(20): 6843–6848. 24828638 10.1523/JNEUROSCI.4990-13.2014 PMC6608114

[43] Puschner B, Schacht J (1997) Energy metabolism in cochlear outer hair cells in vitro. Hear Res 114(1-2): 102–106. 9447924 10.1016/s0378-5955(97)00163-9

[44] Mahapatra S, Marcantoni A, Zuccotti A, Carabelli V, Carbone E (2012) Equal sensitivity of Cav1.2 and Cav1.3 channels to the opposing modulations of PKA and PKG in mouse chromaffin cells. J Physiol. 2012 590(20): 5053–73. 10.1113/jphysiol.2012.236729 PMC3497563 22826131

[45] Johnson SL, Eckrich T, Kuhn S, Zampini V, Franz C, et al (2011) Position-dependent patterning of spontaneous action potentials in immature cochlear inner hair cells. Nat Neurosci 14(6): 711–717. 21572434 10.1038/nn.2803 PMC3103712

[46] Allen JB (1986) Measurement of eardrum acoustic impedance. In: Allen JB, Hall JL, Hubbard A, Neely ST and Tubis A (ed) Peripheral Auditory Mechanisms, Springer-Verlag, New York, pp 44–51.

[47] Goutman JD1, Glowatzki E (2007) Time course and calcium dependence of transmitter release at a single ribbon synapse. Proc Natl Acad Sci USA 104(41): 16341–16346. 17911259 10.1073/pnas.0705756104 PMC2042208

[48] Wong AB, Rutherford MA, Gabrielaitis M, Pangrsic T, Göttfert F, et al (2014) Developmental refinement of hair cell synapses tightens the coupling of Ca^2+^ influx to exocytosis. EMBO J 33(3): 247–264. 24442635 10.1002/embj.201387110 PMC3989618

[49] Moore DR (1991) Anatomy and physiology of binaural hearing. Audiology 30(3): 125–34. 1953442 10.3109/00206099109072878

[50] McAlpine D (2005) Creating a sense of auditory space. J Physiol 566: 21–28. 15760940 10.1113/jphysiol.2005.083113 PMC1464715

[51] Trussell LO (1997) Cellular mechanisms for preservation of timing in central auditory pathways. Current Opinion in Neurobiology 7: 487–492. 9287194 10.1016/s0959-4388(97)80027-x

[52] Bollmann JH, Sakmann B, Borst JG (2000) Calcium sensitivity of glutamate release in a calyx-type terminal. Science 289: 953–957. 10937999 10.1126/science.289.5481.953

[53] Schneggenburger R, Neher E (2000) Intracellular calcium dependence of transmitter release rates at a fast central synapse. Nature 406: 889–893. 10972290 10.1038/35022702

[54] Goutman JD (2012) Transmitter release from cochlear hair cells is phase locked to cyclic stimuli of different intensities and frequencies. J Neurosci 32: 17025–17035. 23175853 10.1523/JNEUROSCI.0457-12.2012 PMC3705563

[55] Li GL, Cho S, von Gersdorff H (2014) Phase-locking precision is enhanced by multiquantal release at an auditory hair cell ribbon synapse. Neuron 83(6): 1404–1417. 25199707 10.1016/j.neuron.2014.08.027 PMC4209920

[56] Rose JE, Brugge JF, Anderson DJ, Hind JE (1967) Phase-locked response to low-frequency tones in single auditory nerve fibers of the squirrel monkey. J Neurophysiol 30: 769–793. 4962851 10.1152/jn.1967.30.4.769

[57] Cho S, Li GL, von Gersdorff H (2011) Recovery from short-term depression and facilitation is ultrafast and Ca2+ dependent at auditory hair cell synapses. J Neurosci 31(15): 5682–5692. 21490209 10.1523/JNEUROSCI.5453-10.2011 PMC3090423

[58] Johnson SL, Kennedy HJ, Holley MC, Fettiplace R, Marcotti W (2012) The resting transducer current drives spontaneous activity in prehearing mammalian cochlear inner hair cells. J Neurosci 32(31): 10479–10483. 22855797 10.1523/JNEUROSCI.0803-12.2012 PMC3428842

[59] Müller M (1996) The cochlear place-frequency map of the adult and developing Mongolian gerbil. Hear Res 94: 148–156. 8789820 10.1016/0378-5955(95)00230-8

[60] Johnson SL, Forge A, Knipper M, Münkner S, Marcotti W (2008) Tonotopic variation in the calcium dependence of neurotransmitter release and vesicle pool replenishment at mammalian auditory ribbon synapses. J Neurosci 28: 7670–7678. 18650343 10.1523/JNEUROSCI.0785-08.2008 PMC2516938

[61] Heidrych P, Zimmermann U, Kuhn S, Franz C, Engel J, et al (2009) Otoferlin interacts with myosin VI: implications for maintenance of the basolateral synaptic structure of the inner hair cell. Hum Mol Genet 18: 2779–2790. 19417007 10.1093/hmg/ddp213

[62] McManus OB, Blatz AL, Magleby KL (1987) Sampling, log binning, fitting, and plotting durations of open and shut intervals from single channels and the effects of noise. Pfluegers Arch 410: 530–553. 2448743 10.1007/BF00586537

[63] Colquhoun D, Hawkes AG (1987) A note on correlations in single ion channel records. Proc R Soc Lond B Biol Sci 230: 15–52. 2438700 10.1098/rspb.1987.0008

